# Supplementary material for: Biodiversity of Actinomycetes from Heavy Metal Contaminated Technosols
Source: Microorganisms. 2021 Jul 30;9(8):1635. doi: 10.3390/microorganisms9081635 (PMC8401206; doi:10.3390/microorganisms9081635)
Supplement: Supplementary file 1 [file microorganisms-09-01635-s001.zip › microorganisms-1302146-supplementary.pdf]

Supplementary Table S1. List of streptomycete strains from heavy metal contaminated areas.

Streptomyces isolate	Strain	Accession number	Clade [1]	Isolation site	Country	Resistance	Literature
S. sp.	EK-I92	GU935327	x	Farmland near Sereď	SK	?	[2]
S. sp.	EK-I89	GU935324	40			?	
S. sp.	K11	JX982494	x	Žiar nad Hronom disposal site		Zn	[3]
S. sp.	M3	AY741284	103	Wastewater of a copper filter plant	AR	Cr	[4]
S. sp.	M40	AY741285	103				
S. sp.	M46	AY741286	x				
S. sp.	AB2A	AY741363	75	Copper polluted sediments, Tucumán	AR	Cu	[5,6]
S. sp.	AB3	AY741364	100				
S. sp.	AB5A	EF527810	80				
S. sp.	F4	DQ141201	103	Former uranium mine, Thuringia	DE	Cd	[7]
S. sp.	P5	AF401982	39	Closed gold mines, Cheon-an and Kong-ju	KR	Pb	[8]
S. sp.	C1	AF403713	29				
S. sp.	R22	AF331829	46	Polluted areas in the Salí River	AR	Cr	[9]
S. sp.	R25	AF331830	?				
S. sp.	CHR28	AF026081	?	Metal-contaminated sediments from Baltimore Inner Harbor	USA	Hg	[10]
S. sp.	CHR3	AF026080	103				
S. sp.	M4	?	?	Anthropogenically contaminated soils, Brno city	CZ	Zn, Cu	[11]
S. sp.	ON3	?	?			Zn, Cu	
S. acidiscabies	NB05-2F	FJ546739	5	Polluted site at the former uranium mine, Thuringia	DE	Cu, Cd, Zn, Ni	[12]
S. sp.	P16A-1, P10A-3	?	20	Banks of creeks in above mentioned area, polluted as a consequence of inflow of acid mine drainage water		Ni, Zn	[13,14]
S. sp.	P4B-1, P10A-4	?	114			Ni, Zn	
S. sp.	P6A-1	?	x			Ni, Zn	
S. sp.	P9A-1	?	x			Ni, Zn	
S. sp.	K7A-1	?	20			Ni, Zn	
S. plumbiresistens	CCNW HX 13-160	EU526954	x	Lead-polluted soil, Gansu province	CN	Pb	[15]
S. zinciresistens	CCNW NQ 0016	GU225938	90	Zinc and copper mine, Shaanxi province	CN	Zn	[16,17]
S. thermocarboxydus	NH50	AJ249627	109	Soil contaminated with metal finishing effluents, Rhône-Alpes	FR	Cr	[18]
S. sp.	BN2, BN9, BN, 12, BN23, BN69, BN71, SB22	KF479164, KF479168, KF479169, KF479178, KF479173, KF479179, KF479188	126	Abandoned mining areas, Marrakech	MR	Pb, Zn, Cr, Cd	[19]

S. sp.	BN3, BN4, BN24, BN68, BN72	KF479165, KF479167, KF479166, KF479174, KF479177, KF479180	112				
S. sp.	BN7, BN13, BN17, BN22, BN25, BN48	KF479170, KF479171, KF479172, KF479175, KF479176	113				
S. sp.	BN73	KF479181	119				
S. sp.	BN82	KF479182	x				
S. sp.	GT1, GT2	KF479183, KF479184,	26				
S. sp.	SB30, SB31	KF479189, KF479190	x				
S. griseorubens	BUCBT-09	KP228016	100	Soil contaminated with pesticides and heavy metals, Santiago del Estero	AR	Cr	[20,21]
S. sp.	H-KF8	KT799850	6	Marine sediments of the Comau Fjord with the natural occurrence of heavy metals, Northern Patagonia	CL	Cu, Co, Hg, Cr, Ni and Te	[22,23]
S. sporoverrucosus	dwc-3	KC508633.1	39	Disposal site for (ultra-) low uraniferous radioactive waste	CN	-	[24]
S. cyaneochromogenes	MK-45	NR_170501.1	x	Manganese-contaminated area, Xiangtan	CN	-	[25]
S. sp.	CdTB01	NZ_CP013743.1	x	Soil contaminated with heavy metals in Xiangxi Tujia and Miao, Hunan Province	CN	Cu, Cr, Cd, Zn	[26]
S. cadmiisoli	ZFG47	NR_171522.1	x	Soil in a cadmium-contaminated area in Xiangtan City, Hunan Province	CN	-	[27]
S. xiangtanensis	LUSFXJ	NR_164877.1	x	Manganese-polluted soil, Xiangtan Manganese Mine	CN	-	[28]
S. manganisoli	MK 44	KY911452.1	x			-	[29]
S. ciscaucasicus	CCNW HX 72-14	EU744543	8	Lead zinc mine tailing	CN	Zn, Cu, Cd	[30]

x = not in the study of the authors Labeda *et al*. [1],
? = no data

References

1. Labeda, D. P.; Goodfellow, M.; Brown, R.; Ward, A. C.; Lanoot, B.; Vanncanneyt, M.; Swings, J.; Kim, S.-B.; Liu, Z.; Chun, J.; Tamura, T.; Oguchi, A.; Kikuchi, T.; Kikuchi, H.; Nishii, T.; Tsuji, K.; Yamaguchi, Y.; Tase, A.; Takahashi, M.; Sakane, T.; Suzuki, K. I.; Hatano, K. Phylogenetic Study of the Species within the Family Streptomycetaceae. *Antonie van Leeuwenhoek* **2012**, *101*, 73–104, doi:10.1007/s10482-011-9656-0.

2. Karelová, E.; Harichová, J.; Stojnev, T.; Pangallo, D.; Ferianc, P. The Isolation of Heavy-Metal Resistant Culturable Bacteria and Resistance Determinants from a Heavy-Metal-Contaminated Site. *Biologia* **2011**, *66*, 18–26, doi:10.2478/s11756-010-0145-0.

3. Sedlakova-Kadukova, J.; Kopcakova, A.; Gresakova, L.; Godany, A.; Pristas, P. Bioaccumulation and Biosorption of Zinc by a Novel Streptomyces K11 Strain Isolated from Highly Alkaline Aluminium Brown Mud Disposal Site. *Ecotoxicol Environ Saf.* **2019**, *167*, 204–211, doi:10.1016/j.ecoenv.2018.09.123.

4. Polti, M. A.; Amoroso, M. J.; Abate, C. M. Chromium(VI) Resistance and Removal by Actinomycete Strains Isolated from Sediments. *Chemosphere* **2007**, *67*, 660–667, doi:10.1016/j.chemosphere.2006.11.008.

5. Albarracín, V. H.; Amoroso, M. J.; Abate, C. M. Isolation and Characterization of Indigenous Copper-Resistant Actinomycete Strains. *Geochemistry* **2005**, *65*, 145–156, doi:10.1016/j.chemer.2005.06.004.

6. Albarracín, V. H.; Ávila, A. L.; Amoroso, M. J.; Abate, C. M. Copper Removal Ability by *Streptomyces* Strains with Dissimilar Growth Patterns and Endowed with Cupric Reductase Activity. *FEMS Microbiol. Lett.* **2008**, *288*, 141–148, doi:10.1111/j.1574-6968.2008.01335.x.

7. Siñeriz, M. L.; Kothe, E.; Abate, C. M. Cadmium Biosorption by Streptomyces Sp. F4 Isolated from Former Uranium Mine. *J. Basic Microbiol.* **2009**, *49*, 55–62, doi:10.1002/jobm.200700376.

8. Rho, J.-Y.; Kim, J.-H. Heavy Metal Biosorption and Its Significance to Metal Tolerance of Streptomycetes. *J. Microbiol.* **2002**, *40*, 51–54.

9. Amoroso, M. J.; Castro, G. R.; Durán, A.; Peraud, O.; Oliver, G.; Hill, R. T. Chromium Accumulation by Two Streptomyces Spp. Isolated from Riverine Sediments. *J Ind Microbiol Biotechnol.* **2001**, *26*, 210–215, doi:10.1038/sj.jim.7000112.

10. Ravel, J.; Amoroso, M. J.; Colwell, R. R.; Hill, R. T. Mercury-Resistant Actinomycetes from the Chesapeake Bay. *FEMS Microbiol. Lett.* **1998**, *162*, 177–184, doi:10.1111/j.1574-6968.1998.tb12996.x.

11. Majzlik, P.; Strasky, A.; Adam, V.; Nemec, M.; Trnkova, L.; Zehnalek, J.; Hubalek, J.; Provaznik, I.; Kizek, R. Influence of Zinc(II) and Copper(II) Ions on Streptomyces Bacteria Revealed by Electrochemistry. *Int. J. Electrochem. Sci.* **2011**, *6*, 2171–2191.

12. Schmidt, A.; Schmidt, A.; Haferburg, G.; Kothe, E. Superoxide Dismutases of Heavy Metal Resistant Streptomycetes. *J Basic Microbiol.* **2007**, *47*, 56–62, doi:10.1002/jobm.200610213.

13. Schmidt, A.; Haferburg, G.; Schmidt, A.; Lischke, U.; Merten, D.; Ghergel, F.; Büchel, G.; Kothe, E. Heavy Metal Resistance to the Extreme: Streptomyces Strains from a Former Uranium Mining Area. *Geochemistry* **2009**, *69*, 35–44, doi:10.1016/j.chemer.2007.11.002.

14. Schmidt, A.; Haferburg, G.; Sineriz, M.; Merten, D.; Büchel, G.; Kothe, E. Heavy Metal Resistance Mechanisms in Actinobacteria for Survival in AMD Contaminated Soils. *Geochemistry* **2005**, *65*, 131–144, doi:10.1016/j.chemer.2005.06.006.

15. Guo, J. K.; Lin, Y. B.; Zhao, M. L.; Sun, R.; Wang, T. T.; Tang, M.; Wei, G. H. Streptomyces Plumbiresistens Sp. Nov., a Lead-Resistant Actinomycete Isolated from Lead-Polluted Soil in North-West China. *Int J Syst Evol Microbiol.* **2009**, *59*, 1326–1330, doi:10.1099/ijs.0.004713-0.

16. Lin, Y.; Wang, X.; Wang, B.; Mohamad, O.; Wei, G. Bioaccumulation Characterization of Zinc and Cadmium by Streptomyces Zinciresistens, a Novel Actinomycete. *Ecotoxicol Environ Saf.* **2012**, *77*, 7–17, doi:10.1016/j.ecoenv.2011.09.016.

17. Lin, Y. B.; Wang, X. Y.; Li, H. F.; Wang, N. N.; Wang, H. X.; Tang, M.; Wei, G.-H. Streptomyces Zinciresistens Sp. Nov., a Zinc-Resistant Actinomycete Isolated from Soil from a Copper and Zinc Mine. *Int J Syst Evol Microbiol.* **2011**, *61*, 616–620, doi:10.1099/ijs.0.024018-0.

18. Desjardin, V.; Bayard, R.; Huck, N.; Manceau, A.; Gourdon, R. Effect of Microbial Activity on the Mobility of Chromium in Soils. *Waste Management* **2002**, *22*, 195–200, doi:10.1016/S0956-053X(01)00069-1.

19. El Baz, S.; Baz, M.; Barakate, M.; Hassani, L.; el Gharmali, A.; Imziln, B. Resistance to and Accumulation of Heavy Metals by Actinobacteria Isolated from Abandoned Mining Areas. *ScientificWorldJournal.* **2015**, *2015*, doi:10.1155/2015/761834.

20. Dávila Costa, J. S.; Hoskisson, P. A.; Paterlini, P.; Romero, C. M.; Alvarez, A. Whole Genome Sequence of the Multi-Resistant Plant Growth-Promoting Bacteria Streptomyces Sp. Z38 with Potential Application in Agroindustry and Bio-Nanotechnology. *Genomics* **2020**, *112*, 4684–4689, doi:10.1016/j.ygeno.2020.08.022.

21. Simón Solá, M. Z.; Lovaisa, N.; Dávila Costa, J. S.; Benimeli, C. S.; Polti, M. A.; Alvarez, A. Multi-Resistant Plant Growth-Promoting Actinobacteria and Plant Root Exudates Influence Cr(VI) and Lindane Dissipation. *Chemosphere* **2019**, *222*, 679–687, doi:10.1016/j.chemosphere.2019.01.197.

22. Undabarrena, A.; Ugalde, J. A.; Seeger, M.; Cámara, B. Genomic Data Mining of the Marine Actinobacteria *Streptomyces* Sp. H-KF8 Unveils Insights into Multi-Stress Related Genes and Metabolic Pathways Involved in Antimicrobial Synthesis. *PeerJ* **2017**, *5*, 1–35, doi:10.7717/peerj.2912.

23. Undabarrena, A.; Beltrametti, F.; Claverías, F. P.; González, M.; Moore, E. R. B.; Seeger, M.; Cámara, B. Exploring the Diversity and Antimicrobial Potential of Marine Actinobacteria from the Comau Fjord in Northern Patagonia, Chile. *Front Microbiol.* **2016**, *7*, doi:10.3389/fmicb.2016.01135.

24. Li, X.; Ding, C.; Liao, J.; Du, L.; Sun, Q.; Yang, J.; Yang, Y.; Zhang, D.; Tang, J.; Liu, N. Bioaccumulation Characterization of Uranium by a Novel Streptomyces Sporoverrucosus Dwc-3. *J Environ Sci (China).* **2016**, *41*, 162–171, doi:10.1016/j.jes.2015.06.007.

25. Tang, X.; Zhao, J.; Li, K.; Chen, Z.; Sun, Y.; Gao, J. Streptomyces Cyaneochromogenes Sp. Nov., a Blue Pigment-Producing Actinomycete from Manganese-Contaminated Soil. *Int J Syst Evol Microbiol.* **2019**, *69*, 2202–2207, doi:10.1099/ijsem.0.003406.

26. Zhou, G.; Yang, H.; Zhou, H.; Wang, C.; Fu, F.; Yu, Y.; Lu, X.; Tian, Y. Complete Genome Sequence of the Streptomyces Sp. Strain CdTB01, a Bacterium Tolerant to Cadmium. *J Biotechnol.* **2016**, *229*, 42–43, doi:10.1016/j.jbiotec.2016.04.040.

27. Li, K.; Tang, X.; Zhao, J.; Guo, Y.; Tang, Y.; Gao, J. Streptomyces Cadmiisoli Sp. Nov., a Novel Actinomycete Isolated from Cadmium-Contaminated Soil. *Int J Syst Evol Microbiol.* **2019**, *69*, 1024–1029, doi:10.1099/ijsem.0.003262.

28. Mo, P.; Yu, Y.-Z.; Zhao, J.-R.; Gao, J. Streptomyces Xiangtanensis Sp. Nov., Isolated from a Manganese-Contaminated Soil. *Antonie van Leeuwenhoek* **2017**, *110*, 297–304, doi:10.1007/s10482-016-0797-z.

29. Mo, P.; Zhao, J.; Li, K.; Tang, X.; Gao, J. Streptomyces Manganisoli Sp. Nov., a Novel Actinomycete Isolated from Manganese-Contaminated Soil. *Int J Syst Evol Microbiol.* **2018**, *68*, 1890–1895, doi:10.1099/ijsem.0.002762.

30. Li, H.; Lin, Y.; Guan, W.; Chang, J.; Xu, L.; Guo, J.; Wei, G. Biosorption of Zn(II) by Live and Dead Cells of Streptomyces Ciscaucasicus Strain CCNWHX 72-14. *J Hazard Mater.* **2010**, *179*, 151–159, doi:10.1016/j.jhazmat.2010.02.072.
